# Towards an In-Depth Understanding of Physical Activity and Eating Behaviours during COVID-19 Social Confinement: A Combined Approach from a Portuguese National Survey

**DOI:** 10.3390/nu13082685

**Published:** 2021-08-02

**Authors:** Marlene Nunes Silva, Maria João Gregório, Rute Santos, Adilson Marques, Bruno Rodrigues, Cristina Godinho, Catarina Santos Silva, Romeu Mendes, Pedro Graça, Miguel Arriaga, Graça Freitas

**Affiliations:** 1 Programa Nacional para a Promoção da Atividade Física, Direção-Geral da Saúde, 1049 Lisboa, Portugal; rsantos.ciafel@fade.up.pt (R.S.); amarques@fmh.ulisboa.pt (A.M.); brunocrodrigues94@gmail.com (B.R.); godinhocristina@gmail.com (C.G.); catarinasantosilva@dgs.min-saude.pt (C.S.S.); romeuduartemendes@gmail.com (R.M.); 2 CIDEFES, Faculdade de Educação Física e Desporto, Universidade Lusófona de Humanidades e Tecnologias, 1749 Lisboa, Portugal; 3 Programa Nacional para a Promoção da Alimentação Saudável, Direção-Geral da Saúde, 1049 Lisboa, Portugal; mariajoaobg@dgs.min-saude.pt; 4 Faculdade de Ciências da Nutrição e Alimentação da Universidade do Porto, 4150 Porto, Portugal; pedrograca@fcna.up.pt; 5 Research Centre in Physical Activity, Health and Leisure, Faculty of Sport, University of Porto, 4200 Porto, Portugal; 6 Laboratory for Integrative and Translational Research in Population Health (ITR), 4050 Porto, Portugal; 7 CIPER, Faculdade de Motricidade Humana, Universidade de Lisboa, 1495 Lisboa, Portugal; 8 Católica Research Centre for Psychological, Family and Social Wellbeing, Universidade Católica Portuguesa, 1649 Lisbon, Portugal; 9 EPIUnit-Instituto de Saúde Pública, Universidade do Porto, 4050 Porto, Portugal; 10 Northern Region Health Administration, 4000 Porto, Portugal; 11 Divisão de Literacia, Saúde e Bem-Estar, Direção-Geral da Saúde, 1049 Lisboa, Portugal; miguelarriaga@dgs.min-saude.pt; 12 Direção-Geral da Saúde, 1049 Lisboa, Portugal; gracafreitas@dgs.min-saude.pt

**Keywords:** physical activity, sedentary behaviour, eating behaviour, socio-demographic correlates, health and risk patterns, COVID-19 social confinement

## Abstract

Rapid worldwide decreases in physical activity (PA), an increase in sedentary behaviour (SB) and poorer dietary patterns have been reported during COVID-19 confinement periods. However, as national variability has been observed, this study sought to describe PA, SB and eating patterns, and to explore their gender as well as other socio-demographic correlates and how they interrelate in a representative sample of Portuguese adults during the COVID-19 first mandatory social confinement. The survey was applied online and by telephone to 5856 adults (mean age = 45.8 years; 42.6% women). The majority reported high (46.0%) or moderate (20.5%) PA levels. Men, younger participants, those with higher education levels and a favourable perception of their financial situation reported higher PA levels, with the opposite pattern for SB. Physical fitness activities and household chores were more reported by women, with more strength training and running activities reported by men. Regarding eating behaviours, 45.1% reported changes, positive (58%) and negative (42%), with 18.2% reporting increases in consumption of fruit, vegetables, and fish and other seafood consumption, while 10.8% (most with lower educational level and less comfortable with their income) reported an increase in consumption of ready-to-eat meals, soft drinks, savoury snacks, and take-away and delivered meals. Two clusters—a health-enhancing vs. risky pattern—emerged through multiple correspondence analysis characterized by co-occurrence of high vs. low PA levels, positive vs. negative eating changes, awareness or not of the COVID-19 PA and dietary recommendations, perceived financial situation, higher vs. lower educational level and time in social confinement. In conclusion, while in social confinement, both positive and negative PA and eating behaviours and trends were displayed, highlighting the role of key sociodemographic correlates contributing to healthy vs. risky patterns. Results may inform future health interventions and policies to be more targeted to those at risk, and also advocate the promotion of PA and healthy eating in an integrated fashion.

## 1. Introduction

The coronavirus disease 2019 (COVID-19) pandemic has placed an overwhelming burden on health systems and authorities to respond with effective and appropriate policies, health communication, and interventions [1,2]. To tackle this pandemic, nonpharmacological, behavioural interventions became mandatory to reduce disease transmission and associated morbidity and mortality [1,3,4]. Furthermore, restaurants, gyms, parks, community centres, and other public social and recreational facilities and venues were closed or their access restricted in many countries, depending on the epidemiologic evolution of the disease, whereas several professionals and students transitioned to online work and learning, respectively, during the state of emergency declared in several countries. However, these nonpharmacological interventions, although necessary to curtail the spread of the disease, potentially disrupted many regular aspects of life, including physical activity (PA) and eating habits [5,6,7,8,9], with potentially critical implications for the global burden of disease [10,11,12,13,14].

Several longitudinal studies have documented the association between social isolation and increased risk of chronic illnesses and mortality, independently of other sociodemographic factors or pre-existing health conditions [14,15,16,17,18,19]. One pathway proposed to underpin such relationship is that socially isolated individuals may adopt less favourable lifestyles, such as poor diets or decreased PA levels [20,21,22]. Thus, adequate eating and PA are considered interconnected strategic public health priorities during this pandemic [1,2,14,21,22].

### 1.1. Social Confinement and Eating Behaviours

Anxiety and boredom evoked by social confinement at home are considered risk factors for dysfunctional eating behaviours, both in terms of “emotional eating”, characterized by overeating of poor-quality foods, and in terms of restricted food access, compared to standard living conditions [6,23]. Furthermore, restricted access to food suppliers due to lockdown and social confinement has placed a burden on regular food-related behaviours [6,24]. This is noteworthy, as maintaining an adequate nutritional status is important for health and well-being, particularly when the immune system is challenged [25]. Malnutrition (both undernutrition and overweight) seems to be a risk factor for poor outcomes in COVID-19 patients [26]. Additionally, limited access to fresh food could negatively affect overall health [24,25]. Thus, it is paramount for researchers and health authorities to identify alterations to dietary patterns during the pandemic, and their socio-demographic correlates [20]. Furthermore, combined with the potential for lower PA levels, impaired dietary habits may lead to a positive energy balance (i.e., weight gain), another critical public health problem [27].

### 1.2. Social Confinement and Physical Activity

Regular PA is important for the immune system and to counteract many comorbidities, such as obesity, diabetes, hypertension, and heart conditions, that increase susceptibility to severe COVID-19 illness [28,29,30,31], as there is a linear relationship between physical activity levels and immune function/viral defense [32,33]. Recommended amounts of 150 min of moderate-intensity or 75 min of vigorous-intensity PA per week [34,35] are consistent with enhanced immunosurveillance and lowered risk for respiratory illness via effects on the immune, respiratory, cardiovascular, and musculoskeletal systems (for a comprehensive review please see [7]). Thus, PA is a relevant adjuvant to COVID-19 mitigation practices [5,36,37] to be promoted as much as social distancing actions [7,8,9,38,39].

In this line, the World Health Organization encouraged governments to include clear exceptions for PA in nationwide lockdowns, allowing outdoor individual PA, provided that adequate interpersonal distance could be maintained. Furthermore, a wide range of exercises, such as video, or app-guided, equipment free, aerobics or strength training to be performed at home were also encouraged [2,11,40]. Nonetheless, social isolation seems to negatively impact PA and sedentary behaviour (SB) levels and patterns, by limiting participation in usual daily activities, travel and access to other forms of exercise (e.g., gyms, parks, and other recreational facilities) [41,42,43,44,45,46,47]. However, some individuals also show maintained, or even increased, levels of PA while in confinement (e.g., more available time, no other competing tasks, as possible way to enjoy the outdoors) [41,48,49,50].

### 1.3. The Need for Adequate, Local, and Integrated Monitoring—A Step for Effective Public Health Policies

Overall, epidemiological studies on the COVID-19 pandemic have indicated a decrease in PA levels [6,41,42,44,45,46,47,51] and nutritional quality [6], although highlighting the heterogeneity of results across countries and even within the same country [41,47,51,52]. Indeed, the extent to which lifestyle behaviours are being impacted by the current COVID-19 pandemic is most likely linked to the stringency of individual government confinement policies and contextual and cultural contingencies. Furthermore, it is of the utmost importance to understand the role of gender, especially considering that policy responses have not yet addressed gender impacts of disease outbreaks [53]. Along with gender differences, other socio-economic indicators should also be considered [53]. These include access to specific COVID-19 public health guidelines (including PA and healthy eating) and their perceived usefulness [54,55].

In sum, in spite of population studies on dietary and PA habits already published in response to this much-needed surveillance effort [21], most surveys did not provide a comprehensive analysis of the PA and diet interactions and common determinants. Indeed, a matter worthy of further consideration is the synergetic effect of PA and eating behaviours [20,56]. For example, low PA levels have been suggested to interact with appetite dysregulation, whereas adequate PA levels seem to relate to a better regulation of eating behaviours [57]. Taking all these factors into consideration will allow identifying potential health and risk patterns, facilitating better tailored future public health recommendations.

In this context, The REACT-COVID survey aimed to (i) describe PA, SB and eating habits, and (ii) explore their gender as well as other socio-demographic correlates and how they interrelate, in a representative sample of Portuguese adults during the COVID-19 pandemic initial critical period (national emergency state conductive to mandatory social confinement at home, in 2020).

## 2. Methods

### 2.1. Study Design and Participants

The REACT-COVID survey was a cross-sectional observational study. Portuguese adults, aged 16 years and older, living in social confinement (as imposed by national authorities throughout the second trimester of 2020), were invited to participate in the survey. Data were collected between 9 April and 4 May 2020, through a nonprobabilistic sampling procedure. Recruitment of participants followed two strategies: a request to participate in the survey was advertised in institutional websites, media and virtual social networks (snowball sampling) and, complementarily, a telephone survey was conducted with owners of fixed and mobile telephone numbers using a Computer-Assisted Telephone Interviewing (CATI) system. The telephone numbers were randomly generated, ensuring a scattered national geographical distribution. After a maximum of five unsuccessful contact attempts at different times of the day and different days of the week, the telephone numbers were considered as nonresponsive. Specific training regarding interviewing techniques, and focused on the questions of the survey, was given to the health professionals (nutritionists and psychologists) who conducted the telephone interviews. The complementary telephone interviews were conducted to ensure increased response rates for socioeconomic groups that are usually underrepresented in online surveys. The LimeSurvey^®^ platform was used to register the answers to both web-based self-administrated questionnaire and interviewer administrated data collection.

The strategies for survey communication and dissemination were adjusted throughout the fieldwork period, according to the collected sociodemographic database profile, targeting an adequate representation of all sociodemographic subgroups, considering sex, decennial group ages, having or not university-level studies and distribution by residence in the Portuguese regions.

Before data collection, the REACT-COVID protocol was submitted and approved by the Ethics Committee of the Centro Académico de Medicina de Lisboa. The study procedures complied with the Declaration of Helsinki for observational studies. Participants were informed about the aims and procedures before enrolment, either by telephone, or via the online study information sheet that accompanied the survey. There were no financial incentives for participation in the study.

### 2.2. The Survey

The questionnaire included indicators about PA, food-related behaviours, perceived reasons for changing these behaviours in the context of social confinement, and access to (and perception of the usefulness of) public health guidelines on PA and healthy eating behaviours during the confinement period. Participants were also asked about details of confinement (e.g., duration and reason for confinement—quarantine or prophylactic isolation; self-perceived general health status), as well as about sociodemographic characteristics (gender, age, educational level, residential municipality, having children or not in the household, and subjective financial well-being). The questionnaire was designed to be completed in about 15 min to maximize participation and the quality of the answers.

#### 2.2.1. Eating Behaviours

Regarding eating behaviours, participants were asked whether they had modified their pattern of food intake compared with the pre-confinement period. This was asked for a set of foods: vegetables, fruit, meat, fish and seafood, sweet snacks (e.g., cookies, biscuits, or chocolates), savoury snacks (e.g., crisps), ready meals (e.g., pizza or lasagne), canned seafood, other canned food (not seafood), take-away or delivered meals, fruit juices, soft drinks, water, and alcoholic beverages. For each of these food groups, participants indicated if they diminished, maintained, or increased their intake. The questionnaire included a checklist of possible reasons for the reported changes in food intake. Participants were also asked to indicate their food shopping habits during the confinement period, and an overall perception regarding the quality of their current (during confinement) diet. Finally, two questions addressed food insecurity.

#### 2.2.2. Physical Activity and Sedentary Behaviour

PA and SB were assessed with the International Physical Activity Questionnaire (IPAQ) [58,59]. Participants reported the frequency and duration of total vigorous and moderate PA and walking performed in 10 min bouts over the previous week, as well as the time spent in SB (sitting time) on a usual weekday. Total weekly duration of PA and energy expenditure of PA were calculated according to the IPAQ scoring protocol, by weighting the time reported for each activity intensity with its estimated metabolic equivalent (MET) energy expenditure [59]. Participants were categorized as having a Health-Enhancing PA (HEPA), active and low active, according to IPAQ scoring references [59,60]. Participants were also classified according to three categories of time spent sitting (SB), according to tertiles: 0–180 min a day; 181–419 min a day; ≥420 min a day [61].

### 2.3. Statistical Analyses

Data cleaning procedures considered the completion time of the questionnaire. For that, the number of answers and completion times were used to calculate mean time of response for the total questionnaire and for the last group of answers of the questionnaire. Five seconds was established as an acceptable minimum completion time average per question. Respondents whose average completion time per question was lower than this were excluded from the analysis.

A minimum sample size of 400 individuals per NUT II region of Portugal (North, Center, Lisbon Metropolitan Area, Alentejo, Algarve, Autonomous Region of the Madeira, and Autonomous Region of the Azores) was estimated to be adequate for the planned analysis. This corresponds to an overall minimum sample size of 2800 individuals.

To verify the representativeness of the sample according to the Portuguese population, we first compared the socioeconomic characteristics of surveyed sample with the Portuguese population distribution. Information of the Census 2011 was used for weighing the sample based on the distributions of sex (men and women), age group (16–34; 35–54; and 55 years or more), level of education (secondary level or lower; and higher education) and NUTS II (North, Center, Lisbon Metropolitan Area, Alentejo, Algarve, Autonomous Region of the Madeira, and Autonomous Region of the Azores) for the Portuguese population. Weighted absolute frequencies (and corresponding proportions) were provided for categorical variables and continuous variables were described by weighted mean values and SDs. Participants who had missing data in variables used for weighing the sample were excluded from analyses.

All statistics were performed using IBM SPSS Statistics^®^ (version 26). The mean time of response for the total questionnaire was calculated, and a cut-off for an acceptable minimum completion time was then established. Responses whose completion time was lower than the established cut-off were excluded from the analyses. Age was categorized in age groups (16–34; 35–54; and 55 years or more) and the level of education was dichotomised in secondary school or below vs. higher education. Participants’ characteristics were described using weighted absolute frequencies (and corresponding percentages). Food intake and PA-related variables were described by main sociodemographic characteristics (gender, age groups, region, financial situation). Tests of independence were performed using the Chi-Square test or Fisher’s Exact Tests (according to the number of cells and variable distribution). To understand how subjects could be nested within clusters of health-enhancing vs. risky patterns of PA and eating behaviours and related variables, Multiple Correspondence Analysis (MCA) was used to explore the data and produce a simplified graphic representation of the information by analysing cases-by-variable categories matrices. Prior to this analysis, which combines patterns of PA and eating behaviours, MCA and Agglomerative Hierarchical Cluster analysis (AHC) were used to identify patterns of changes on food consumption behaviour during the home confinement, compared to before the lockdown.

## 3. Results

### 3.1. Participants’ Characteristics

Table 1 depicts participants’ characteristics. The sample comprised 5856 adults aged 16 and older (mean age 45.8 years). Most participants had secondary or higher education (81.5% and 18.5%, respectively). The majority of the participants were employed or studying (82%) and without children (67.8%); 43.6% were in-home social confinement for more than 4 weeks at the time of the survey, and the most common reasons reported for being in social confinement were mandatory teleworking (30.3%) and family support (16.6%).

### 3.2. Physical Activity and Sedentary Behaviour

Table 2 presents the prevalence of PA and SB by gender, age, education, perception of the family financial situation, knowledge and self-rated usefulness of the COVID-19 PA recommendations, and time spent in home social confinement. During social confinement, 46% of the participants (49.9% men and 42.6% women, *p* > 0.001) were classified as HEPA. As age increased, the number of participants classified as HEPA decreased significantly. Participants with higher education levels were more frequently classified as HEPA.

Regarding the time spent sitting, 37.1% of the participants reported spending at least, 420 min per day (7 h) in this behaviour, with men, younger participants, those with higher education levels, those reporting a more favourable perception of the family financial situation, and those who did not know the PA recommendation spending significantly more time sitting in social confinement.

Table 3 summarizes the type of PA and SB engaged during social confinement. Among men and women, the most frequently reported physical activities during social confinement were house cleaning (men: 51.1%; women: 86.7%, *p* > 0.001), climbing up and down stairs (men: 49.3; women 50.6%), and walking (men: 31.5%; women: 32.9%). Watching television (men: 70.6%; women: 69.5%), using the computer, the tablet or a smartphone (men: 66.5%; women: 55.4%, *p* < 0.001), and teleworking (men: 39%; women: 34.4%, *p* > 0.05) were the most frequently reported sedentary activities.

### 3.3. Eating Habits

**Figure 1:**
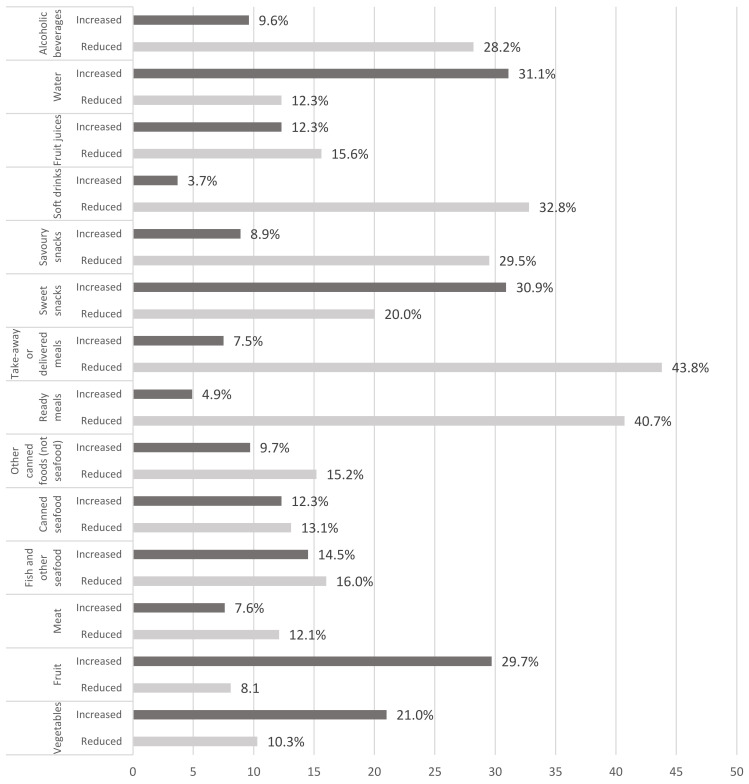
Change in food intake during COVID-19 confinement.

In terms of eating habits, almost half of the participants (45.1%) reported general changes in their eating habits during social confinement and 37.8% reported changes in diet quality (58.1% improved and 41.9% worsened) (Table 4). Women (47.3% vs. 42.6% in men, *p* < 0.001), young people (57.1% in individuals aged 16–34 years vs. 33.1% in individuals aged more than 55 years, *p* < 0.001), and those with high educational level (49.1% vs. 44.2%, *p* < 0.05) were more likely to report changes of eating habits during the COVID-19 confinement. The reported changes in food consumption are presented in Figure 1 and Table 5. Sweet snacks (30.9%), fruit (29.7%), and vegetables (21.0%) were the food categories with the highest proportion of a reported increase in consumption during social confinement, with women reporting the highest increase in vegetables (22.1% vs. 19.9%, *p* < 0.05) and sweet snacks (32.8% vs. 28.8%, *p* < 0.05) consumption, and men the highest increase in fruit consumption (32.2% vs. 27.4%, *p* < 0.001). From the food categories with the highest proportion of a reported decrease in consumption, we highlight takeaway or delivered meals (43.8%), ready meals (40.7%), soft drinks (32.8%), savoury snacks (30.9%), and alcoholic beverages (28.2%). When we combine and analyze together the different food items, two main “dietary patterns” were identified. One “healthy food behaviour pattern” that combines an increase in, at least, two of the following favourable foods (fruit, vegetables, and fish) and an “unhealthy food behaviour pattern” characterized by an increase in ready meals, savoury snacks, soft drinks and take-away consumption, and by a decrease in fruit and vegetable consumption (Table 5). The “unhealthy food behaviour pattern” was more likely to be followed by men (*p* < 0.001), younger groups (*p* < 0.001), and by those individuals reporting living with a less comfortable income (*p* < 0.05) and at risk of food insecurity (*p* < 0.001). During the analysed period, the estimated proportion of the risk of food insecurity was 33.2%, and 8% reported facing economic difficulties in accessing food.

Table 4 presents other changes in food consumption behaviour during the confinement period. Cooking more (56.9%) and a decrease in shopping frequency (87.3%) were the most reported changes. An increase in snacking between meals was also reported by 31.4% of the participants (Table 4).

Weight change was also reported by a significant proportion of participants. In this study, 21.0% and 12.9% of the participants reported an increase and decrease in body weight, respectively (Table 4).

The most frequently reported reasons for changes in food consumption during COVID-19 social confinement can be grouped into four main drivers: the need to reduce the frequency of shopping (34.3%); changes in appetite (changes in appetite in general (19.3%) and changes in appetite caused due to stress (18.6%)); changes in daily routines (different work schedule (17.6%) and changes on regular place for shopping (10.6%)), and changes related with the economic context (10.3%). The main reasons for changes in food consumption during COVID-19 social confinement seemed to be different according to gender. Changes in appetite in general (20.5% vs. 18.0%, *p* < 0.05) and changes in appetite caused by stress (19.5% vs. 15.9%, *p* < 0.001) were more frequently reported by women, while changes in daily routines (different work schedule) were more frequently reported by men (19.5% vs. 15.9%, *p* < 0.001) (Table 6).

### 3.4. Combined Approach: Health and Risk Patterns

**Figure 2:**
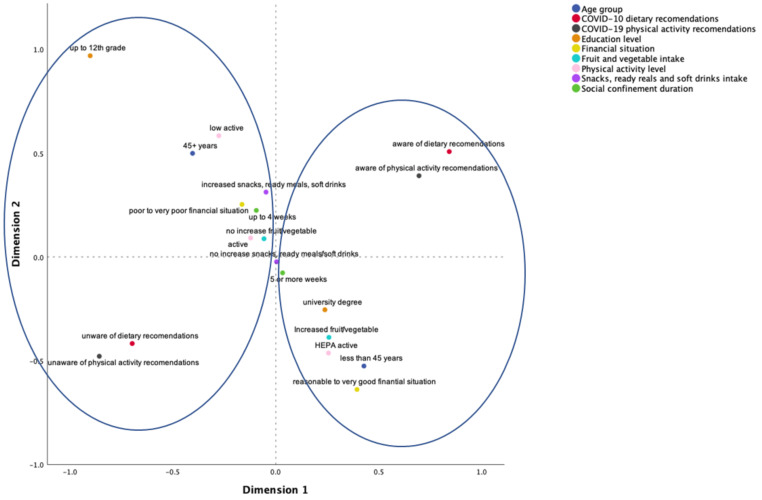
Health vs. risk patterns.

The MCA yielded two dimensions with eigenvalues of 1.715 and 1.344, respectively, and inertia of 0.191 (i.e., explaining 19.1% of the variance) and 0.149 (i.e., explaining 14.9% of the variance). The model explained 34% of the variance (inertia = 0.340, with an Eigenvalue of 3.059). From this analysis, two patterns were identified: a risky pattern and a healthy pattern (herein called clusters). Figure 2 displays the MCA results, showing the two clusters. The first cluster corresponds to a health-enhancing pattern with the co-occurrence of higher PA levels; increased fruit and vegetable consumption; awareness and knowledge of the COVID-19 PA and dietary recommendations; perceived financial situation as reasonable, good, or very good; higher educational level, as well as being for a shorter period of time in social confinement (up to 3 weeks). The second cluster is compatible with a risky pattern, pointing to the co-occurrence of low PA levels; increased consumption of savoury snacks, ready-to-eat meals, soft drinks, takeaways and decreased consumption of fruit and vegetables; not knowing about the COVID-19 PA and dietary recommendations; perceived financial situation as difficult or very difficult; lower educational level, as well as being for a longer period of time in social confinement (4 weeks or more).

## 4. Discussion

This study sought to characterize PA and SB levels and changes in food behaviours and explore gender and other socio-demographic correlates, including a combined analysis of the health behaviours surveyed and their common determinants, in Portuguese adults. It was carried out during the first wave of the COVID-19 pandemic after governmental actions imposing social confinement and limiting participation in normal daily activities and routines.

Regarding PA levels, the results of this survey showed that the majority of the population surveyed reported HEPA (46.0% vs. 27.1% in 2017 IAN-AF [62]) and moderate PA levels (33.5% vs. 30.3% in 2017 IAN-AF [62]) during social confinement. Only 20.5% reported low PA levels (vs. 42.6% in 2017 IAN-AF [62]). These results conflict with other international surveys that described decreases in PA levels during confinement periods [41,42,44,45,46,47], and align with the literature pointing to an increase [49,50] and remarkable variations from country to country [51], highlighting the need for specific, comprehensive, national surveillance.

It is important to note that this increase in the prevalence of adequate HEPA levels did not occur homogeneously. Several influences need to be taken into consideration, namely the effects of (i) gender (42.6% of women were classified as HEPA vs. almost 50% men); (ii) age (as age decreased, the number of participants classified as HEPA increased significantly; and (iii) socioeconomic status (those with higher education and good perceived financial situation were more frequently classified as HEPA). Regarding SB, these influences also apply, although in a different direction: men, younger participants, those with higher education levels and with a favourable perception of the family financial situation, reported spending significantly more time sitting. Given these different influences (opposite risk directions), it might be that the promotion of behaviour change may have to follow different strategies and targets when it comes to reducing sitting time or increasing PA, especially in social-confinement contexts.

In this last regard, gender differences were also visible in the type of structured physical activities undertaken. Although walking was the most frequently reported PA in both genders, women significantly reported more physical fitness activities, whereas men reported more frequently being involved in strength training and running activities. For nonstructured PA, domestic activities and climbing up and down the stairs was expressively indicated by all participants inquired. Still, marked gender disparities were observed concerning domestic activities/household chores, with women being the ones that carried out these activities the most. Thus, it would be of paramount importance to further address the role of gender, especially considering that policy responses have not yet addressed the gendered impacts of disease outbreaks [63].

Concerning eating behaviours, this study showed that a high proportion of the population surveyed changed their eating habits during the confinement period of the first wave of the COVID-19 pandemic (45.1%), 58% of those reported an improvement in their diet (main positive changes reported: increase in fruit, vegetables, and water consumption and a decrease in savoury snacks, ready-to-eat meals, soft drinks, and alcoholic beverages intake), while 42% reported a worsening tendency, namely an increased consumption of sweet snacks. This data supports previous findings that both favorable and unfavorable changes in dietary habits were observed during the COVID-19 confinement period [64,65,66,67,68,69]. The confinement period had immediate effects on individuals’ lives, altering daily living routines, imposing food shopping restrictions (lower frequency), and worsening stress load [70]. All these factors seem to have impacted eating habits. However, for 18.2% of the participants, an improvement in eating habits was reported, with a combined increase in fruit, vegetables and fish and other seafood consumption, and more time spent cooking. Contrarily, a worsened dietary pattern was found for 10.8% of the population surveyed in our study, characterized by a combined increase in ready-to-eat meals, soft drinks, savoury snacks, and take-away and delivered meals. These findings are in line with a narrative synthesis [71] of the trajectories of food choices during the COVID-19 confinement periods that included 12 studies and showed an initial decline in diet quality. In general, diet was characterized by an increase in the consumption of carbohydrates sources and snacking. However, the results of this narrative review also found positive eating changes, such as a slightly increased consumption of fruit and vegetables and an important increase in the habit of cooking meals at home.

Moreover, our results also suggest that the COVID-19 pandemic might have increased the social gap in dietary quality. Individuals that live with a less comfortable income tend to have higher adherence to worsened dietary patterns. This survey’s results also allowed a more in-depth understanding of the main reasons pointed out for eating behaviours. Three main triggers were reported: (i) disruption in daily routines and work; (ii) changes in appetite due to anxiety feelings and stress; (iii) the economic uncertainty in terms of future access to food. These triggers were unequally distributed by gender. Disruption of work and daily routines was the most reported driver for men, while changes in appetite due to anxiety feelings and stress were more frequently reported by women.

Regarding the impact of the COVID-19 lockdown on eating habits, it is important to consider the economic damage effects on food insecurity. Data from our study shows a high percentage of individuals at risk of food insecurity (33.2%), and 8% are facing economic difficulties in accessing food. This prevalence is higher when compared to data from 2015/2016 (19.3% of Portuguese households living with food insecurity) [72,73].

Most importantly, and considering the potential interactional nature of the variables under analysis in this study (and that changes in one domain may aggregate with changes in others [56,57]), it is important to note that a combined approach, via MCA, highlighted the emergence of clusters of risk and health protection factors, suggesting, as in previous studies, that health-enhancing spill-over effects may arise when being more physically active and having a healthier eating pattern co-occur [74,75]. Furthermore, our results show that this protective behavioural pattern also clusters with a good perceived financial situation, higher educational level and awareness and knowledge of the COVID-19 PA and dietary recommendations for social confinement, launched by the Portuguese Directorate-General of Health’ Eating and PA National Promotion Programs. These findings are in line with the assertion that, more than a pandemic, COVID-19 may be a syndemic [76], inviting a larger vision, encompassing education, employment, housing, food, and environment, and recognizing how political and social factors drive, perpetuate, or worsen the emergence and clustering of diseases. Indeed, our second cluster further points to the co-occurrence of low PA levels: increased consumption of savoury snacks, soft drinks, take-aways, and decreased consumption of fruit and vegetables; not knowing about the COVID-19 PA and dietary recommendations; perceiving the financial situation as difficult or very difficult; lower educational level, as well as being for a longer period in social confinement (4 weeks or more).

### Strengths and Limitations

This cross-sectional survey involved a large sample size that, after being weighted, is similar to the Portuguese residents’ population concerning sociodemographic characteristics. To overcome the constraints of pure online administration (only reaching a certain socio-demographic profile), phone interviews with the same set of questions were also conducted. Survey contents were based on self-report measures. Although device-based measures would be preferred due to their accuracy, the public health measures related to the pandemic precluded their use. Furthermore, device-based measures may not capture domain-and context-specific PA behaviours, essential for this study’s aims. Thus, according to best practices, the survey was based on widely used, validated methods to measure PA and SB [37]. Nonetheless, overestimation of PA may occur when IPAQ is used. In the domain of eating behaviours, an adapted self-report set of questions was also created. Following what has been advocated to conduct studies pertaining to a behavioural epidemiology framework [37], there was an effort in balancing the need for precision vs. feasibility (i.e., necessary adaptations for collecting data during the pandemic). The main analyses conducted in this study allowed us to describe the main PA and eating behaviours during a critical time (first wave of the pandemic-related social confinement), while also exploring their correlates and aggregation patterns, a much needed research endeavor given that, and despite a new burst of studies in this regard, the correlates of lifestyle behaviours (e.g., eating, PA, and SB) and their interactions remain under-researched [22], and international surveys have pointed to national and regional disparities that need to be further explored in the face of national and local public health policies and jurisdiction [6,37,51]. Longitudinal follow-ups will be needed to determine the long-term impact of pandemic-related public health measures on these critical health behaviours.

## 5. Conclusions

In Portugal, acknowledging the exceptions allowing for outdoor exercise, and also an increased offer of PA guidance and apps and all sorts of exercise classes available on social media, results indicate a positive scenario when compared to previous national surveys: raising the numbers of those in adequate HEPA levels and lowering the ones with low levels. The preferences expressed by the population surveyed may be informative for future health campaigns, highlighting not only the preference for walking and fitness activities, but also the role of informal activities as an intentional way of being active (e.g., house cleaning, climbing up and down stairs).

Concerning eating indicators, an increasing trend in perceived food insecurity was noticed when compared to previous surveys, a finding warranting the attention of public authorities. Changes in dietary habits occurred in positive and negative ways, and two patterns of eating behaviours emerged, suggesting that bad eating habits (e.g., increase savoury snacks, soft drinks, and take-away consumption and decrease in fruit and vegetable consumption) vs. good eating habits foods (e.g., fruit, vegetables, and fish) tend to cluster.

Nonetheless, all results need to be understood considering the role of gender, as significant differences emerged, and overall women tended to show impaired PA levels, stress-induced eating and different PA and eating patterns and preferences which need to be taken in consideration. Furthermore, age, socio-economic status, health literacy and time in social confinement were also correlates of the results, contributing to different health and risk patterns, highlighting: (i) the need for future interventions and public health policies to be more targeted to reach those most in need, in particular the most vulnerable groups, at-risk groups (older adults, socio-economically impaired persons, more time in social confinement); and (ii) the potential advantage, for future interventions and policies, of promoting PA and healthy eating simultaneously and in an integrated fashion, as positive spill-over effects may apply facilitating co-occurrence.

## Figures and Tables

**Table 1:** Participants’ characteristics of the Portuguese REACT COVID-19 survey.

Participants’ Characteristics	Total	Men	Women	*p*
	% (95% CI)	% (95% CI)	% (95% CI)	
**Age**	45.8 (45.4, 46.3)	45.5 (44.9, 46.1)	46.2 (45.6, 46.8)	
**Age-group**				<0.001
16–34 years	27.9 (26.8, 29.1)	29.5 (27.8, 31.2)	26.4 (24.8, 27.9)	
35–54 years	34.9 (33.7, 36.1)	35.9 (34.2, 37.7)	34.0 (32.3, 35.7)	
≥55 years	37.2 (35.9, 38.4)	34.5 (32.7, 36.3)	39.6 (37.9, 41.3)	
**Education**				<0.001
Secondary school or below	81.5 (80.5, 82.5)	83.5 (82.1, 84.9)	79.9 (78.5, 81.4)	
Higher education	18.5 (17.5, 19.5)	16.5 (15.1, 17.9)	20.1 (18.6, 21.5)	
**Professional situation**				<0.001
Employee	57.4 (56.1, 58.8)	58.7 (56.8, 60.6)	56.2 (54.4, 58.0)	
Student	15.1 (14.1, 16.0)	14.9 (13.5, 16.3)	15.2 (13.8, 16.5)	
Retired	17.4 (16.4, 18.4)	19.3 (17.8, 20.9)	15.7 (14.3, 17.0)	
Housekeeper	3.4 (2.9, 3.8)	0.1 (0.0, 0.2)	6.4 (5.5, 7.3)	
Unemployed	6.7 (6.1, 7.4)	6.9 (6.0, 7.9)	6.6 (5.7, 7.5)	
**Subjective financial well-being**				0.004
Comfortable or very comfortable	30.1 (28.9, 31.3)	30.3 (28.6, 32.1)	29.9 (28.3, 31.6)	
Reasonable	51.3 (50.0, 52.6)	52.8 (50.9, 54.7)	49.8 (48.1, 51.6)	
Difficult or very difficult	18.6 (17.6, 19.7)	16.8 (15.4, 18.3)	20.2 (18.8, 21.7)	
**Children**				0.448
No	67.8 (66.6, 69.0)	67.3 (65.5, 69.1)	68.2 (66.6, 69.9)	
Yes	32.2 (31.0, 33.4)	32.7 (30.9, 34.5)	31.8 (30.1, 33.4)	
**Social confinement (reasons)**				
Sickness (COVID-19)	1.2 (1.0, 1.5)	1.9 (1.4, 2.4)	0.7 (0.4, 1.0)	<0.001
Sickness (other)	2.2 (1.8, 2.6)	1.3 (0.9, 1.8)	3.0 (2.4, 3.6)	<0.001
Family support	16.6 (15.6, 17.6)	14.3 (12.9, 15.6)	18.7 (17.3, 20.1)	0.064
Telework	30.3 (29.1, 31.5)	31.5 (29.8, 33.2)	29.3 (27.7, 30.9)	<0.001
Prophylactic isolation	5.5 (4.9, 6.1)	7.9 (6.9, 9.0)	3.3 (2.7, 4.0)	
**Lockdown (time)**				0.246
Up to 3 weeks	26.0 (24.9, 27.1)	26.7 (25.0, 28.3)	25.5 (23.9, 27.0)	
Up to 4 weeks	43.6 (42.3, 44.9)	44.0 (42.1, 45.8)	43.2 (41.5, 44.9)	
Up to 5 weeks	30.3 (29.2, 31.5)	29.4 (27.7, 31.1)	31.3 (29.7, 33.0)	
**Self-rated health**				<0.001
Very good	37.4 (36.2, 38.7)	43.0 (41.2, 44.9)	32.5 (30.8, 34.1)	
Good	37.2 (36.0, 38.4)	35.6 (33.8, 37.4)	38.7 (36.9, 40.4)	
Reasonable	25.4 (24.3, 26.5)	21.4 (19.9, 22.9)	28.9 (27.3, 30.5)	

Weighed data: age (*n* = 5856), education (*n* = 5856), professional situation (*n* = 5532), family income perception (*n* = 5643), children (*n* = 5856), time of lockdown (*n* = 5856), self-rated health (*n* = 5856). Differences between men and women were tested by Chi-Square.

**Table 2:** Prevalence of physical activity and time spent sitting, during the COVID-19 social confinement.

Participants’ Characteristics	IPAQ Categories			*p*	Sitting Time (min/day)			*p*
	% (95% CI)				% (95% CI)			
	Low Active	Active	HEPA		<180	181–419	≥420	
Total	33.5 (32.3, 34.7)	20.5 (19.5, 21.5)	46.0 (44.7, 47.2)		34.4 (33.2, 35.7)	28.4 (27.3, 29.6)	37.1 (35.9, 38.4)	
**Gender**				<0.001				<0.001
Men	29.4 (27.7, 31.1)	20.7 (19.2, 22.2)	49.9 (48.0, 51.7)		29.8 (28.1, 31.5)	30.8 (29.1, 32.5)	39.4 (37.5, 41.2)	
Women	37.1 (35.4, 38.8)	20.3 (18.9, 21.7)	42.6 (40.9, 44.4)		38.5 (36.7, 40.2)	26.4 (24.8, 27.9)	35.2 (33.5, 36.8)	
**Age-group**				<0.001				<0.001
16–34 years	27.5 (25.3, 29.6)	18.6 (16.7, 20.5)	53.9 (51.5, 56.3)		24.4 (22.3, 26.4)	26.1 (24.0, 28.3)	49.6 (47.1, 52.0)	
35–54 years	37.0 (34.9, 39.1)	16.3 (14.7, 17.9)	46.6 (44.4, 48.8)		36.0 (33.9, 38.1)	29.0 (27.0, 31.0)	35.0 (32.9, 37.0)	
≥55 years	34.8 (32.8, 36.8)	25.8 (24.0, 27.7)	39.4 (37.3, 41.4)		40.6 (38.5, 42.6)	29.6 (27.7, 31.5)	29.8 (27.8, 31.7)	
**Education**				<0.001				<0.001
Secondary school or below	34.6 (33.3, 36.0)	20.8 (19.7, 22.0)	44.6 (43.1, 46.0)		37.4 (36.0, 38.8)	28.9 (27.6, 30.1)	33.7 (32.4, 35.1)	
Higher education	28.7 (26.0, 31.4)	19.1 (16.8, 21.4)	52.2 (49.2, 55.2)		21.4 (19.0, 23.8)	26.7 (24.0, 29.3)	52.0 (49.1, 55.0)	
**Family income perception**				<0.001				<0.001
Good	25.6 (23.5, 27.6)	21.7 (19.8, 23.7)	52.7 (50.3, 55.1)		33.7 (31.5, 36.0)	26.4 (24.3, 28.5)	39.9 (37.5, 42.2)	
Reasonable	34.9 (33.2, 36.6)	21.7 (20.2, 23.2)	43.4 (41.6, 45.3)		31.4 (29.7, 33.1)	29.1 (27.5, 30.8)	39.5 (37.7, 41.3)	
Poor	40.8 (37.8, 43.7)	16.9 (14.7, 19.2)	42.3 (39.3, 45.3)		39.7 (36.8, 42.7)	29.8 (27.0, 32.5)	30.5 (27.7, 33.3)	
**PA recommendation knowledge**				<0.001				0.234
Yes	34.8 (33.0, 36.6)	19.5 (18.0, 21.0)	45.7 (43.8, 47.6)		37.6 (35.7, 39.4)	27.5 (25.8, 29.2)	34.9 (33.1, 36.7)	
No	32.5 (30.8, 34.1)	21.3 (19.9, 22.7)	46.3 (44.5, 48.0)		31.8 (30.2, 33.4)	29.2 (27.6, 30.8)	38.9 (37.2, 40.6)	
**PA usefulness of recommendations**				<0.001				<0.001
Not useful	36.2 (30.9, 41.5)	13.7 (9.9, 17.4)	50.2 (44.6, 55.7)		24.4 (19.7, 29.2)	38.1 (32.7, 43.5	37.1 (31.8, 42.5)	
I am not sure	41.7 (35.9, 47.5)	23.0 (18.1, 28.0)	35.3 (29.6, 40.9)		36.3 (30.7, 42.0)	19.1 (14.4, 23.7)	45.0 (39.1, 50.8)	
Very useful	33.7 (31.7, 35.7)	19.9 (18.2, 21.6)	46.4 (44.2, 48.5)		39.7 (37.6, 41.8)	27.0 (25.1, 28.9)	33.3 (31.2, 35.3)	
**Lockdown (time)**				0.005				0.015
Up to 3 weeks	35.5 (33.1, 37.9)	19.0 (17.0, 20.9)	45.5 (43.0, 48.0)		37.9 (35.4, 40.3)	28.1 (25.9, 30.4)	34.1 (31.7, 36.4)	
Up to 4 weeks	31.0 (29.2, 32.8)	21.7 (20.1, 23.3	47.3 (45.4, 49.3)		33.1 (31.3, 34.9)	28.5 (26.7, 30.2)	38.4 (36.5, 40.3)	
Up to 5 weeks	35.6 (33.3, 37.8)	20.1 (18.2, 22.0)	44.3 (42.0, 46.7)		33.4 (31.2, 35.6)	28.7 (26.6, 30.8)	37.9 (35.7, 40.2)	

Weighed data. Abbreviations: IPAQ, International Physical Activity Questionnaire; PA, physical activity; HEPA—Health-enhancing Physical Activity; CI—Confidence Intervals. Differences among IPAQ categories and sitting time intervals were tested by Chi-Square.

**Table 3:** Physical activities and sedentary behaviours, during the COVID-19 social confinement.

Physical Activities and Sedentary Behaviours	Men	Women	*p*
	% (95% CI)	% (95% CI)	
**Types of physical activities**			
House cleaning	51.1 (49.2, 52.9)	86.7 (85.5, 87.9)	<0.001
Up and down stairs	49.3 (47.4, 51.2)	50.6 (48.9, 52.4)	0.294
Walking	31.5 (29.8, 33.3)	32.9 (31.3, 34.6)	0.253
Fitness activities	21.5 (19.9, 23.0)	28.9 (27.3, 30.5)	<0.001
Gardening	21.8 (20.2, 23.3)	23.3 (21.9, 24.8)	0.147
Bricolage	24.0 (22.4, 25.6)	14.2 (13.0, 15.4)	<0.001
Strength training	23.7 (22.1, 25.3)	12.9 (11.7, 14.1)	<0.001
Jogging	22.7 (21.1, 24.2)	6.5 (5.6, 7.3)	<0.001
Static bicycle	11.7 (10.5, 12.9)	5.3 (4.5, 6.0)	<0.001
Dance	2.2 (1.6, 2.7)	10.1 (9.1, 11.2)	<0.001
**Types of sedentary behaviours**			
Watching television	70.6 (68.9, 72.3)	69.5 (67.8, 71.1)	0.359
Computer, tablet, smartphone	66.5 (64.7, 68.2)	55.4 (53.7, 57.2)	<0.001
Telework	39.0 (37.2, 40.8)	34.4 (32.7, 36.1)	<0.001
Read	28.6 (26.9, 30.3)	33.8 (32.2, 35.5)	<0.001
Board games, puzzles, Legos	8.2 (7.2, 9.2)	9.2 (8.2, 10.2)	0.191
Play musical instrument	3.7 (3.0, 4.4)	1.2 (0.8, 1.6)	<0.001

Weighed data. Men (*n* = 2743), women (*n* = 3097). Differences between men and women were tested by Chi-Square.

**Table 4:** Self-reported changes of food behaviours, during COVID-19 social confinement.

Self-Reported Changes of Food Behaviours	Total	Men	Women	*p*
	% (95% CI)	% (95% CI)	% (95% CI)	
**Changes in dietary habits**				<0.001
Yes	45.1 (43.9, 46.4)	42.6 (40.8, 44.5)	47.3 (45.5, 49.0)	
No	54.9 (53.6, 56.1)	57.4 (55.5, 59.2)	52.7 (50.9, 54.4)	
**Changes in diet quality**				0.037
No changes/do not know	62.2 (60.4, 64.0)	63.8 (61.3, 66.5)	60.9 (48.5, 63.4)	
Yes	37.8 (36.5, 39.0)	36.2 (34.4, 38.0)	39.1 (37.4, 40.8)	
Improved (from those who reported changes)	58.1 (56.1, 60.2)	59.7 (56.6, 62.7)	56.9 (54.2, 59.7)	0.193
Worsened (from those who reported changes)	41.9 (39.8, 43.9)	40.3 (37.3, 43.4)	43.1 (40.3, 45.9)	
**Cook more often**				<0.001
Yes	56.9 (55.6, 58.1)	51.8 (49.9, 53.6)	61.4 (59.7, 63.1)	
No	43.1 (41.9, 44.4)	48.2 (46.4, 50.1)	38.6 (36.9, 40.3)	
**Change in the number of meals**				0.007
No	69.8 (68.6, 71.0)	71.6 (69.9, 73.3)	68.4 (66.7, 70.0)	
Yes	30.2 (29.0,31.3)	28.4 (26.7, 30.1)	31.6 (30.0, 33.3)	
Increased (from those who reported changes)	67.8 (65.6, 69.9)	66.5 (63.1, 69.7)	68.8 (65.8, 71.6)	0.328
Decreased (from those who reported changes)	32.2 (30.1, 34.4)	33.5 (30.1, 36.7)	31.2 (28.4, 34.2)	
Snacking more often *				0.186
Yes	31.4 (30.2, 32.6)	32.2/30.5, 34.0)	30.6 (29.0, 32.3)	
No	68.6 (67.4, 69.8)	67.8 (66.0, 69.5)	69.4 (67.7, 71.0)	
**Changes on shopping frequency**				<0.001
Decreased	87.3 (86.3, 88.3)	82.5 (80.7, 84.2)	91.1 (89.9, 92.3)	
Increased	12.7 (11.7, 13.7)	17.5 (15.8, 19.3)	8.9 (7.7, 10.1)	0.317
**Self-reported changes in body weight**				
No changes/do not know	66.1 (63.8, 68.4)	63.4 (60.2, 66.7)	68.4 (65.2, 71.7)	
Increased	21.0 (20.0, 22.0)	22.3 (20.8, 23.9)	19.8 (18.5, 21.3)	
Decreased	12.9 (12.1, 13.8)	14.3 (13.0, 15.6)	11.7 (10.7, 12.9)	
**Knowledge about dietary recommendations for lockdown period**				<0.001
Yes	45.3 (44.0, 46.6)	38.5 (36.7, 40.3)	51.4 (49.6, 53.2)	
No	54.7 (53.4, 56.0)	61.5 (59.7, 63.3)	48.6 (46.8, 50.4)	
**Usefulness of dietary recommendations for lockdown period**				<0.001
None	11.9 (10.7, 13.2)	14.7 (12.7, 17.0)	10.0 (8.6, 11.5)	
I am not sure	10.5 (9.4, 11.7)	9.2 (7.6, 11.0)	11.3 (9.8, 12.9)	
Very useful	77.6 (76.0, 79.2)	76.0 (73.4, 78.5)	78.7 (76.6, 80.6)	

Weighed data. Total (*n* = 5840), men (*n* = 2743), women (*n* = 3097); * Snacking is defined as any food eaten between main meals. Differences between men and women were tested by Chi-Square.

**Table 5:** Variation in food intake during COVID-19 social confinement by gender.

Food Category	Reduced Intake		*p*	Increased Intake		*p*	Maintained Intake	
	Men	Women		Men	Women		Men	Women
	% (95% CI)	% (95% CI)		% (95% CI)	% (95% CI)		% (95% CI)	% (95% CI)
Take-away or delivered meals	43.3 (41.5, 45.2)	44.3 (42.6, 46.1)	0.459	9.8 (8.8, 11.0)	5.5 (4.7, 6.3)	<0.001	46.9 (45.0, 48.7)	50.2 (48.4, 52.0)
Ready meals	40.5 (38.7, 42.4)	40.8 (39.1, 42.6)	0.831	6.7 (5.9, 7.7)	3.3 (2.7, 3.9)	<0.001	52.7 (50.8, 54.6)	55.9 (54.1, 57.6)
Soft drinks	33.5 (31.7, 35.2)	32.2 (30.6, 33.9)	0.328	4.6 (3.8, 5.4)	3.0 (2.5, 3.7)	0.003	62.0 (60.2, 63.8)	64.8 (63.0, 66.4)
Alcoholic beverages	30.3 (28.6, 32.0)	26.4 (24.9, 28.0)	0.001	12.7 (11.6, 14.0)	6.8 (6.0, 7.7)	<0.001	57.0 (55.2, 58.9)	66.8 (65.1, 68.4)
Savoury snacks	28.2 (26.6, 29.9)	30.7 (29.0, 32.3	0.044	9.3 (8.3, 10.4)	8.5 (7.6, 9.5)	0.311	62.5 (60.7, 64.3)	60.8 (59.1, 62.5)
Sweet snacks	19.0 (17.6, 20.5)	20.9 (19.5, 22.3)	0.082	28.8 (27.2, 30.6)	32.8 (31.1, 34.4)	0.001	52.2 (50.3, 54.0)	46.4 (44.6, 48.1)
Fish and other seafood	17.0 (15.7, 18.5)	15.1 (13.8, 16.3)	0.041	15.5 (14.2, 16.9)	13.7 (12.5, 14.9)	0.053	67.5 (65.7, 69.2)	71.2 (69.6, 72.8)
Fruit juices	14.1 (12.8, 15.4)	17.0 (15.7, 18.3)	0.003	12.6 (11.4, 13.9)	12.0 (10.9, 13.2)	0.498	73.2 (71.6, 74.9)	71.0 (69.4, 72.6)
Other canned food (not seafood)	13.8 (12.6, 15.1)	16.4 (15.1, 17.7)	0.006	9.5 (8.5, 10.7)	9.9 (8.8, 10.9)	0.69	76.7 (75.1, 78.3)	73.8 (72.2, 75.3)
Meat	12.5 (11.3, 13.7)	11.8 (10.7, 13.0)	0.47	8.6 (7.6, 9.7)	6.8 (5.9, 7.7)	0.012	78.9 (77.4, 80.5)	81.4 (80.0, 82.7)
Canned seafood	12.0 (10.8, 13.2)	14.1 (12.9, 15.4)	0.018	13.2 (11.9, 14.5)	11.6 (10.5, 12.8)	0.079	74.8 (73.2, 76.4)	74.3 (72.7, 75.8)
Vegetables	10.8 (9.6, 12.0)	9.9 (8.9, 11.0)	0.301	19.9 (18.4, 21.4)	22.1 (20.7, 23.6)	0.04	69.4 (67.6, 71.1)	68.0 (66.3, 69.6)
Water	10.2 (9.1, 11.4)	14.2 (13.0, 15.5)	<0.001	31.4 (29.7, 33.1)	30.8 (29.2, 32.4)	<0.001	58.4 (56.6, 60.3)	55.0 (53.3, 56.8)
Fruit	8.2 (7.2, 9.3)	8.0 (7.1, 9.0)	0.773	32.2 (30.5, 34.0)	27.4 (25.9, 29.0)	<0.001	59.6 (57.8, 61.4)	64.6 (62.9, 66.2)
**Dietary patterns**	**Total; % (95% CI)**			**Men; % (95% CI)**			**Women; % (95% CI)**	***p***
Improved dietary patterns	18.2 (17.2, 19.2)			18.1 (16.7, 19.6)			18.3 (16.9, 19.7)	0.193
Worsened dietary patterns	10.8 (10.1, 11.7)			12.9 (11.7, 14.2)			9.0 (8.0, 10.1)	

Weighed data. Men (*n* = 2743), women (*n* = 3097). Improved dietary patterns represent the increase of consumption of vegetables, fruit and fish. Worsened dietary patterns represent the increase of ready meals, soft drinks, savoury snacks and take-away and delivered meals); Differences between men and women were tested by Chi-Square.

**Table 6:** Reasons for changes in food intake during COVID-19 social confinement.

Reasons for Changes in Food Intake	Total	Men	Women	*p*
	% (95% CI)	% (95% CI)	% (95% CI)	
Changes in shopping frequency	34.3 (33.1, 35.5)	34.8 (33.0, 36.6)	33.9 (32.3, 35.6)	0.469
Changes in appetite	19.3 (18.3, 20.3)	18.0 (16.6, 19.4)	20.5 (19.1, 21.9)	0.015
Changes in appetite caused by stress	18.6 (17.6, 19.6)	14.1 (12.9, 15.5)	22.6 (21.1, 24.0)	<0.001
Different work schedule	17.6 (16.6, 18.6)	19.5 (18.0, 21.0)	15.9 (14.7, 17.2)	<0.001
Changes in regular place for shopping	10.6 (9.9, 11.4)	10.5 (9.4, 11.7)	10.6 (9.6, 11.7)	0.91
Concerns with the economic context	10.3 (9.5, 11.1)	9.9 (8.8, 11.0)	10.6 (9.5, 11.7)	0.391
Different people at meals time	9.7 (9.0, 10.5)	9.8 (8.8, 11.0)	9.7 (8.7, 10.8)	0.873
Problems with access to usually bought food	9.1 (8.4, 9.9)	9.5 (8.5, 10.7)	8.7 (7.8, 9.8)	0.311
Because diet can protect against the novel coronavirus	5.5 (5.0, 6.1)	6.0 (5.2, 7.0)	5.1 (4.3, 5.9)	0.114
Changes on food prices	4.8 (4.3, 5.4)	5.3 (4.5, 6.2)	4.3 (3.7, 5.1)	0.087
Fear of getting infected with the novel coronavirus through food	3.8 (3.3, 4.3)	4.0 (3.4, 4.8)	3.5 (2.9, 4.2)	0.264
Concerns with possible stock rupture of food in supermarkets	2.5 (2.1, 2.9)	3.5 (2.9, 4.3)	1.6 (1.2, 2.0)	<0.001

Weighed data. Total (*n* = 5840), men (*n* = 2743), women (*n* = 3097). Differences between men and women were tested by Chi-Square.

## Data Availability

Data are available from the authors upon reasonable request.
